# “Gender-mainstreaming” in immunization must be inclusive of transgender and gender diverse people

**DOI:** 10.3389/fgwh.2024.1338409

**Published:** 2024-04-12

**Authors:** Harikeerthan Raghuram, Sharin D’souza, Bhakti Ghatole, Satendra Singh, Aqsa Shaikh, Anant Bhan, Sunita Sheel Bandewar

**Affiliations:** ^1^ Initiative for Health Equity Advocacy and Research, Bhopal Hub, Sangath, Bhopal, India; ^2^ Department of Physiology, University College of Medical Sciences, University of Delhi, Delhi, India; ^3^ Department of Community Medicine, Hamdard Institute of Medical Sciences and Research, Jamia Hamdard, New Delhi, India; ^4^ Forum for Medical Ethics and Society, Pune, India; ^5^ Founding Trustee, Vidhayak Trust, Pune, India

**Keywords:** vaccination, gender, transgender, inclusion, health systems

## Introduction

Gender identity is an important personal characteristic that has ramifications in access to the life-saving service of vaccination. “Gender-mainstreaming” of immunization systems focuses on integrating gender-related concerns into different aspects of immunization toward promoting gender equality (1). Toward this, while there has been significant progress in vaccination research, policy reviews and developments, and programmatic guidance, these largely remain confined within the gender binaries. People of diverse gender identities, such as transgender and gender diverse (TGD) individuals, are often invisible in these documents. This indicates a lack of understanding of gender in all its diversity and has potential adverse consequences for access to vaccines among TGD communities. TGD people are those who have “gender identities or expressions that differ from the gender socially attributed to the sex assigned to them at birth” (2). As researchers who are from the TGD community and/or work with the TGD community closely, especially in the immunization space, we argue the need for the inclusion of TGD constituencies as part of “gender-mainstreaming” guidance efforts, making them gender-inclusive in its true sense, in alignment with the realities on the ground.

## Current conversations

Recent studies in vaccination spaces vindicate our observation that gender framing is restricted to binaries. For example, a 2022 commentary in the *Lancet*, although titled “Shifting gender barriers,” spoke primarily about women and their access to COVID-19 vaccines (3). An empirical study published in the *Lancet* on the effects of the COVID-19 pandemic on gender equality also focused on comparisons between women and men only (4). A 2021 review on sex and gender in COVID-19 vaccine research in *Frontiers in Global Women's Health* also restricted its analysis to men and women (5). There are some indications of a changing canvas in these spaces, though; for example, a 2021 commentary in *BMJ Global Health* explicitly calls for and provides a framework for the inclusion of people with diverse gender identities in COVID-19 vaccination research (6).

Similarly, regarding policies and programs, a 2018 report by UNICEF and the Bill and Melinda Gates Foundation entitled *A gender lens to advance equity in immunization* has no mention of TGD communities (7). Likewise, the Gavi website on “gender and immunization” articulates that “Gavi works with countries to address gender-related barriers, and ensure all boys and girls have equal access to vaccines,” indicating that it fails to be gender-inclusive (8). These are more concerning since these organizations form the background for other global health and development policies, such as the United Nations' sustainable development goals (SDGs), and fail to go beyond women and girls as marginalized gender identities.

In recent years, there have been a few changes, although their scope remains limited toward broadening the understanding of gender within vaccination. For example, the WHO's Gender Equality Strategy 2019–2023 mentions “people with diverse gender identities” once in the document in its mission statement, but the rest of the document is primarily about female empowerment (9). The Gavi Alliance Gender Policy (2020) version 3.0 mentions “those with diverse gender identities,” although only in the rationale and definition sections (10). The WHO Behavioral and Social Drivers COVID-19 vaccination survey for adults and health workers (version 1.0) released in 2022 captures non-binary gender identities as a participant demographic. However, the only question related to gender as a social driver in the survey is related to autonomy in decision making with reference to traveling for COVID-19 vaccination (11). Other documents have instead recognized this binary trans-exclusionary perspective as a limitation. For example, in 2021, “Why gender matters: immunization agenda 2030” has a disclaimer stating that “This document is primarily about the gender inequalities that exist between men and women. Addressing the barriers to immunization faced by others who are gender diverse/non-conforming is also important. While not specifically addressed in this document, many of the principles and tools can also be adapted for these groups” (1). An in-depth critical review of the document would inform the extent of such an adaptability.

## Implications and way forward

Excluding TGD communities in vaccination research precludes the possibility of redesigning vaccination services to be TGD-inclusive. Vaccine trials, in particular, should strive to ensure representation of TGD individuals, in the absence of which, knowledge on the safety of vaccines in TGD individuals will remain unknown and therefore unaddressed (12). For example, in our experience, TGD persons have had concerns about the safety of vaccines while taking hormone replacement therapy (HRT), but no studies examined the interaction of COVID-19 vaccines with HRT (12). Such a dearth of representation of TGD individuals in research lead to policies, public health data, and programs devoid of TGD specificities. For this, it will also be important to have data on the number of TGD individuals in our midst. Policy and programs focusing on gender disparities solely through a binary framework risk sustaining and worsening the disparities faced by TGD individuals. Moving away from a gender-neutral vaccination service to making them women-inclusive was a step forward and yet they continue to exclude TGD individuals. Therefore, these issues point toward the need to warrant further efforts to make them inclusive of people of gender diverse identities. The inclusion of TGD individuals is especially important in the context of newer vaccination programs where gender identities play a much bigger role; for example, the HPV vaccine (currently in most contexts being given to adolescent girls and women) is equally important in transmasculine individuals, but they may get excluded as they do not identify as women.

Making considerations of gender in vaccination research, policy developments and programmatic guidance inclusive of TGD persons is vital to achieve truly gender responsive immunization. This is important in light of the clarion call of “Healthcare for All” and “Leaving No One Behind” from key multilateral organizations. Further, it is necessary to meet the mandates under domestic laws, such as The Transgender Persons (Protection of Rights) Act, 2019, in India (13). Finally, efforts toward the inclusion of TGD individuals are particularly important considering emerging immunization programs related to HPV, Monkeypox, and HIV, diseases that can present a greater risk for the TGD community.

Current approaches that purportedly aim to be inclusive by mentioning that the outputs are limited to gender binary analyses remain inadequate and leave behind a marginalized segment in vaccination efforts. Instead, research, policy, and programs for gender-mainstreaming in immunization must be inclusive of TGD communities. Toward this, we urge multilateral organizations, funding bodies, journals, researchers, and policymakers working in the spaces of vaccination and gender to prioritize generating data for TGD inclusion and take this to action for furthering health equity for all genders.
